# The Role of Tumor Inflammatory Microenvironment in Lung Cancer

**DOI:** 10.3389/fphar.2021.688625

**Published:** 2021-05-17

**Authors:** Zhaofeng Tan, Haibin Xue, Yuli Sun, Chuanlong Zhang, Yonglei Song, Yuanfu Qi

**Affiliations:** ^1^ First Clinical Medical College, Shandong University of Traditional Chinese Medicine, Jinan, China; ^2^ Departments of Oncology Affiliated Hospital of Shandong University of Traditional Chinese Medicine, Jinan, China; ^3^ Eighth Medical Center of the General Hospital of the Chinese People’s Liberation Army, Beijing, China

**Keywords:** tumor microenvironment, inflammation, lung cancer, metastasis, immune escape

## Abstract

Lung cancer is the most common and fatal malignant tumor in the world. The tumor microenvironment (TME) is closely related to the occurrence and development of lung cancer, in which the inflammatory microenvironment plays an important role. Inflammatory cells and inflammatory factors in the tumor inflammatory microenvironment promote the activation of the NF-κB and STAT3 inflammatory pathways and the occurrence, development, and metastasis of lung cancer by promoting immune escape, tumor angiogenesis, epithelial–mesenchymal transition, apoptosis, and other mechanisms. Clinical and epidemiological studies have also shown a strong relationship among chronic infection, inflammation, inflammatory microenvironment, and lung cancer. The relationship between inflammation and lung cancer can be better understood through the gradual understanding of the tumor inflammatory microenvironment, which is advantageous to find more therapeutic targets for lung cancer.

## Introduction

Tumor diseases are increasingly becoming a major disease that seriously endangers human health all over the world. Among all tumors, lung cancer has the greatest prevalence and mortality (Barta et al., 2019). Studies on tumor diseases have shown that the tumor microenvironment (TME), which consists of tumor-related cells and stromal cells, interacts with tumor cells and plays an important role in tumor growth, invasion, and metastasis (Huang et al., 2021). With the rise of precision therapy, the importance of the TME is becoming increasingly significant. In chronic inflammatory diseases and smoldering inflammation caused by tumors, inflammation has a great impact on the composition of the TME, especially on the plasticity of tumors and stromal cells (Greten and Grivennikov, 2019). With the deepening of research on lung cancer and the TME, a close relationship between inflammation and lung cancer has been found, and considerable attention has been paid to the role of inflammation and the inflammatory microenvironment in lung cancer. In addition, some reports indicate that the intervention of tumor inflammatory microenvironment can reduce the development of lung cancer in animal models and patients of lung cancer (Caetano et al., 2016; Weichand et al., 2017).

The TME was first proposed by Lord et al., through the tumor model system *in vitro*, the interaction between lymphoid host cells and multicellular spheres was studied, and the effect of host cells on tumor cells was verified (Lord et al., 1979). In recent years, the TME has become one of the fastest-growing areas of cancer research, which is widely considered to be closely related to the occurrence and development of tumors, and is a hot spot in current cancer research. It mainly includes tumor cells, tumor-associated fibroblasts, immune cells, vascular endothelial cells, extracellular matrix (ECM) and its degrading enzymes, various growth factors, and inflammatory factors. Moreover, it has special physical and chemical characteristics (e.g., hypoxia, low pH) (Wang et al., 2016; Wang et al., 2019; Jiang et al., 2020; Zheng et al., 2020; Liu et al., 2021; Song et al., 2021; Zheng et al., 2021). The TME has multiple effects on tumor initiation, development, and progression, and the mechanism is complex. Therefore, in the treatment of tumors and targeted control of cytokines and related factors in the TME, studying the role of the TME and its related molecular mechanisms will provide a new and important target for cancer therapy. This review mainly focused on inflammatory cells and inflammatory factors in the tumor inflammatory microenvironment and the principal mechanisms that govern the effects of inflammation on lung cancer development.

## Inflammation and Lung Cancer: A Multifaceted Link

**FIGURE 1:**
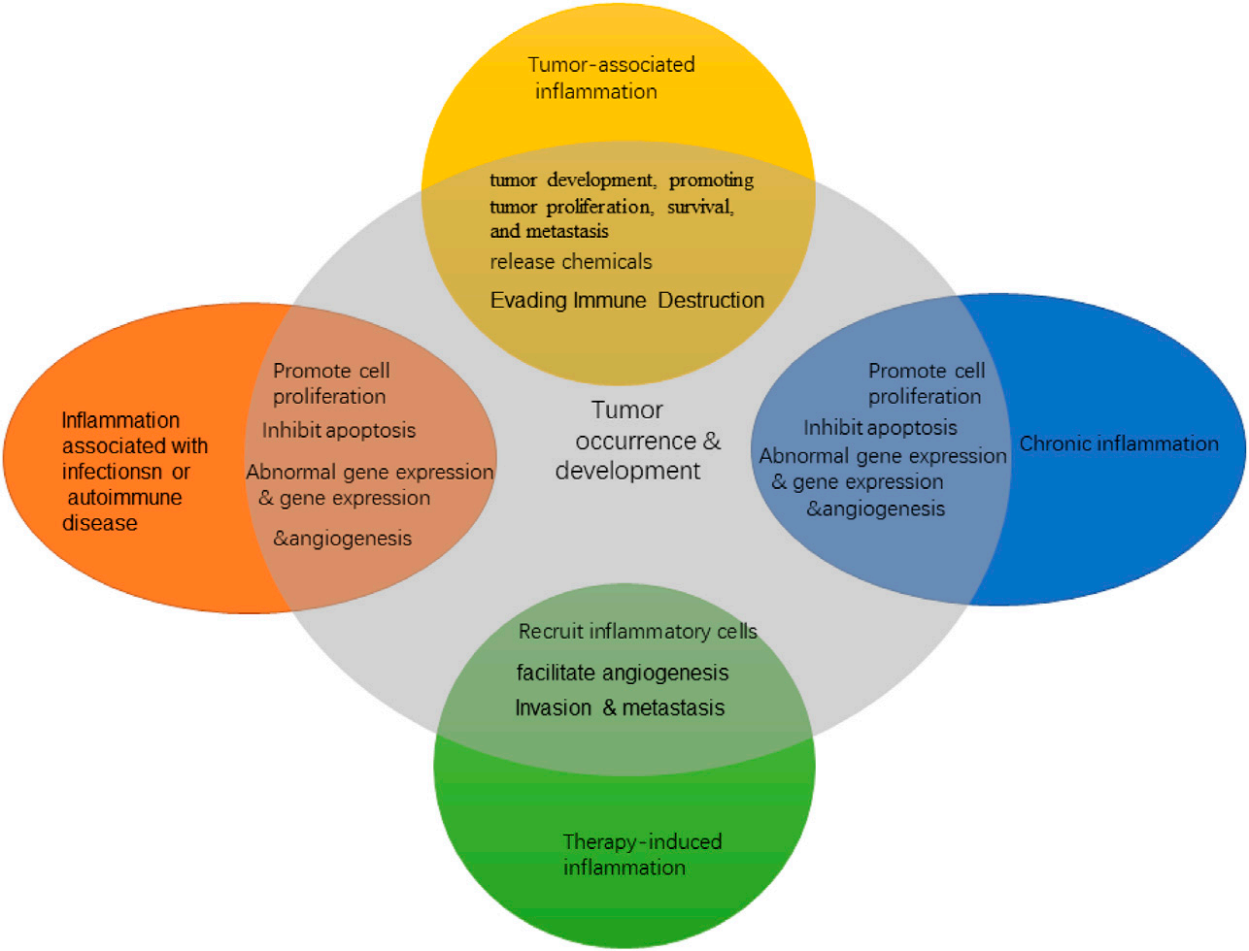
Inflammatory types in cancer.

The persistence of chronic inflammation is one of the important characteristics of malignant tumors. The TME is largely regulated by inflammatory cells and is an indispensable component in tumor development, promoting tumor proliferation, survival, and metastasis. In the 19th century, German pathologist Rudolf Virchow found infiltrating white blood cells in tumor tissue, which suggested for the first time that there may be some relationship between inflammation and tumors. With the development of epidemiology and molecular biology, the close relationship between inflammation and tumors has attracted considerable attention. In 2011, famous cancer scholars Hanahan and Weinberg listed “promoting tumor inflammation” as one of the 10 characteristics of cancer and proposed that inflammation can promote a variety of tumor landmark functions by providing bioactive molecules to the TME (Hanahan and Weinberg, 2011). Inflammation is involved in all stages of tumorigenesis, from malignant transformation and tumor initiation to established tumor invasion and metastasis (Figure 1). Although the mechanism by which inflammation promotes cancer is not fully understood, two interrelated hypotheses have emerged: one is the internal pathway driven by genetic changes that lead to tumors and inflammation; the other is the external pathway driven by inflammatory conditions that increase tumor risk (Conway et al., 2016).

Inflammation promotes the emergence of multiple cancer markers by providing essential molecules to the TME. Several studies have shown that the inflammatory microenvironment may predate tumor formation. Inflammation can lead to tumor formation and development through epigenetics and subsequent abnormal gene expression, vascular abnormalities, and tumor neovascularization and by promoting cell proliferation, inhibiting apoptosis, adjusting genomic stability, and promoting metastasis and other mechanisms. In addition, inflammatory cells can release chemicals such as ROS that promote carcinogenic evolution, and many factors released by inflammatory cells may directly or indirectly lead to significant inhibition of immune response (Hanahan and Weinberg, 2011). Inflammation, especially chronic inflammation, is related to the occurrence and development of various tumors. The inflammatory TME is a common feature of solid tumors. The interaction between inflammatory microenvironment and tumor cells has a profound impact on tumor growth, metastasis, and drug resistance (Kitamura et al., 2015).

Inflammation can easily lead to the development of cancer and promote all stages of tumorigenesis. Clinical and epidemiological studies have shown a strong correlation among chronic infection, inflammation, and lung cancer. Epidemiological studies have shown that people prone to chronic inflammatory diseases have an increased risk of cancer, and potential infections and inflammation are associated with 15–20% of cancer deaths worldwide (Bremnes et al., 2011). The lung is an open organ, where gas exchange occurs. Due to its unique physiological structure, it is easily affected by a large number of pathogens, pollutants, oxidants, gases, and poisons inhaled from the air. Long-term exposure to inhalable silica dust, mineral fibers, air particulate matter, and smoke and chronic inflammation caused by bronchitis, chronic obstructive pulmonary disease (COPD), and bronchial asthma can induce lung cancer (Houghton, 2013; Valavanidis et al., 2013). The vast majority of lung cancer cases are due to continued exposure to tobacco smoke (Gandini et al., 2008). Smoking is the main cause of chronic pulmonary inflammation and lung injury. At the same time, its carcinogens increase the oxidative stress of tissues and cause tissue inflammation. The comprehensive analysis results of Brenner et al. showed that the prevalence of past lung diseases (chronic bronchitis, emphysema, pneumonia, and tuberculosis) independently affects the development of lung cancer in non-smokers and may be related to disease-related inflammation and pathogenesis (Brenner et al., 2012). Inflammation can cause lung tissue damage. During inflammation, the cell division rate, DNA damage, and cell mutation rate in lung tissue are increased. In addition, inflammation increases the likelihood of lung cancer by acting as an initiator or promoter of antiapoptotic signals. It can also cause angiogenesis (the formation of new blood vessels) and provide nutrients for the growth and spread of tumor cells (Azad et al., 2008).

Continuous inflammation will cause a large number of inflammatory cell infiltration, which in turn floods the microenvironment with a large number of inflammatory cytokines and active mediators (such as ROS, TNF-α, etc.) (Azad et al., 2008; Kundu and Surh, 2008). The inflammatory TME plays many roles in tumor progression and metastasis, including creating a hypoxic environment, increasing angiogenesis and invasion, altering the expression of microRNA, and increasing stem cell phenotype, thereby promoting epithelial–mesenchymal transition (EMT) (Heinrich et al., 2012). Hypoxia and decreased tissue oxygen tension are the basic characteristics of the cancer microenvironment. Chronic inflammation also leads to local hypoxia due to the combined effect of reduced circulation at the site of inflammation and increasing metabolic demand for infiltrating immune cells. Hypoxia can lead to increased resistance to conventional radiation and chemotherapy, which further induces the EMT (Semenza, 2010). In non-small cell lung cancer (NSCLC) cell lines, the activation of HIF-1α by cMet stimulates the HGF of tumor cells, which leads to ECM degradation, cell dissociation, and increased cell migration through tissue parenchyma (Yoo et al., 2011). Inflammatory cells in the inflammatory microenvironment can secrete vascular endothelial growth factor (VEGF) in large quantity to promote tumor angiogenesis (Gomes et al., 2014).

Inflammation and the inflammatory microenvironment affect numerous aspects of malignant tumors, including the proliferation and survival of malignant cells, angiogenesis, tumor metastasis, and tumor response to chemotherapy drugs and hormones (Greten and Grivennikov, 2019). In the occurrence of lung cancer, inflammation caused by infection increases the likelihood of lung cancer and affects the prognosis of lung cancer to a certain extent. Preventing and controlling inflammation and finding targets in the inflammatory microenvironment are one of the critical ways to prevent and control lung cancer.

## Inflammatory Cells, Cytokines, and Pathways Associated With Lung Cancer

From the perspective of the TME, the occurrence and progression of lung cancer should be the outcome of problems in the entire body tissue, not just tumor cells. The inflammatory microenvironment is an essential component of the occurrence and metastasis of lung cancer and a key factor in regulating invasion and metastasis.

**FIGURE 2:**
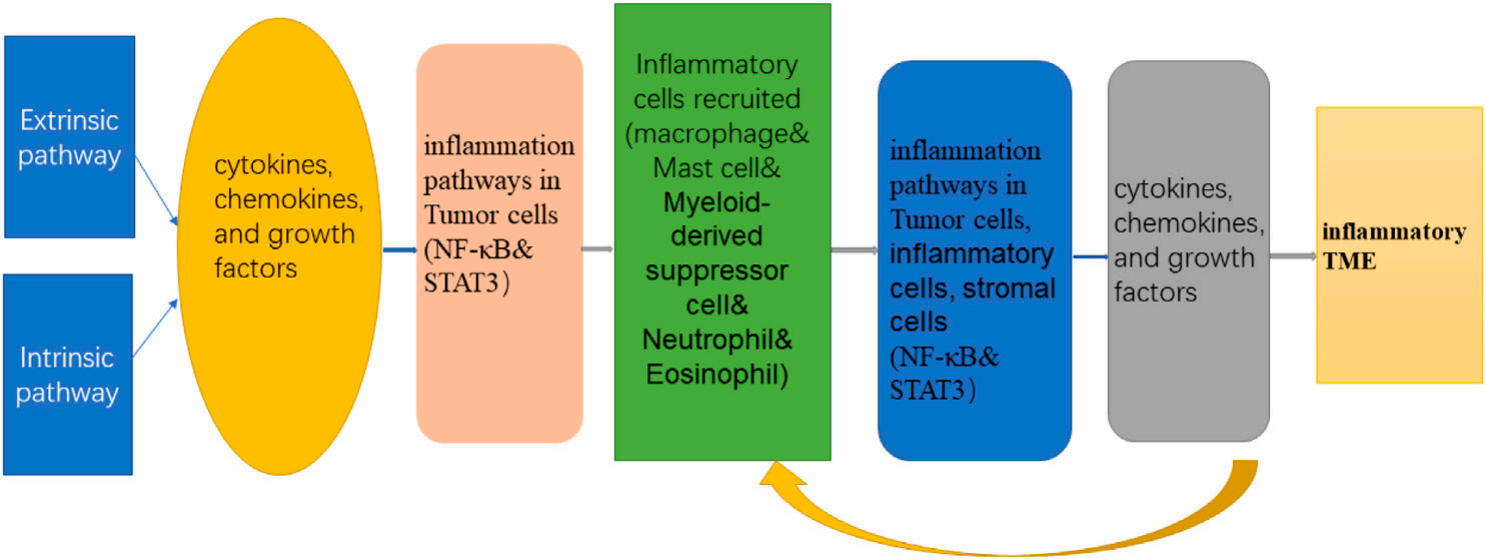
The multifaceted connections of inflammation and cancer.

The neutrophils, macrophages, and myeloid-derived suppressor cells contained in the tumor inflammatory microenvironment and their secreted cytokines, chemokines, and growth factors together affect the progression and metastasis of tumors. Among them, tumor-related cytokines, oxygen metabolites, proteases, inflammatory factors, and other inflammatory mediators can cause mutations in cell genes and induce inflammatory reactions. The inflammatory factors produced in the inflammatory response, such as interleukin (IL-1) and TNF-α, can also promote the activation of NF-κB and STAT3 inflammation pathways, cause damage to cellular genes, induce gene mutations, and ultimately lead to the formation of tumors. At the same time, the activation of NF-κB and STAT3 can increase the production of inflammatory factors, maintain the tumor inflammatory microenvironment, and form a vicious circle of “inflammation–tumor-inflammation” (Figure 2). The following sections mainly introduce the focal inflammatory cells, factors, and inflammatory pathways in the inflammatory microenvironment.

### Inflammatory Cells Associated With Lung Cancer

When the cause of inflammation persists, acute inflammation can be converted into chronic inflammation with significant white blood cell infiltration in the lung tissue. Inflammatory cells associated with lung cancer include T cells, myelogenic suppressor cells, natural killer cells, mast cells, tumor-associated neutrophils (TAN) and macrophages, etc. At present, macrophages are the most studied inflammatory cells.

**FIGURE 3:**
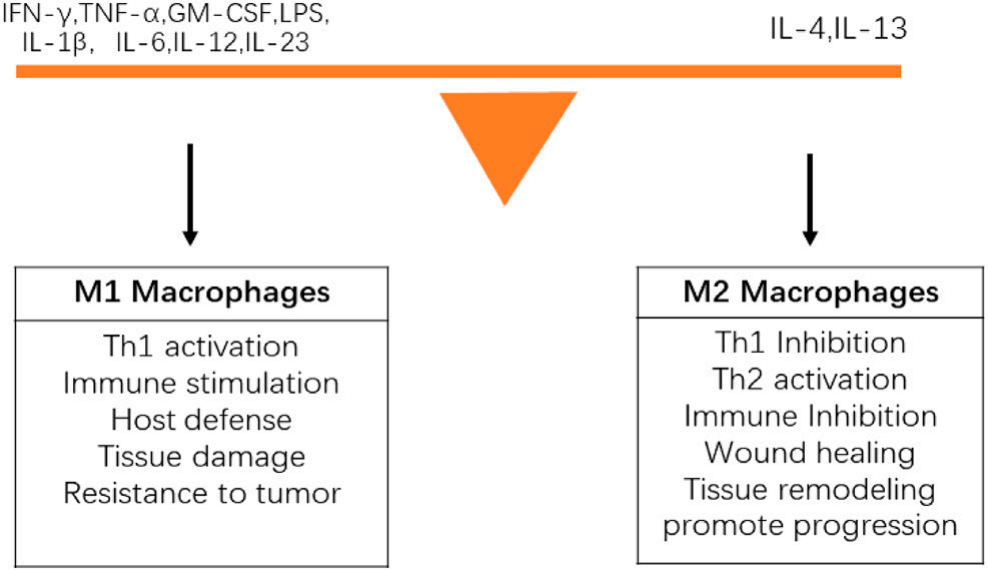
Characteristics of M1 and M2 macrophages.

Macrophages are innate immune cells that not only play a critical role in improving the immune response to foreign cells, bacteria, and viruses, but also mediate tissue repair after injury. Macrophages have various physiological functions, including non-specific immune defense, non-specific immune surveillance, processing and presenting antigens to initiate an adaptive immune response, immunomodulatory effects, and participation in inflammation. Macrophages can participate in and promote inflammation by secreting chemokines and inflammatory factors such as MIP, monocyte chemotactic protein-1 (MCP-1), IL-1, and IL-8. There are at least two types of tumor-associated macrophages (TAMs). (I) Classic M1 macrophages have the following characteristics: efficient antigen presentation function; massive secretion of IL-12 and IL-23; and ability to activate Th1 immune response, eliminate infectious microorganisms, and kill tumor cells. The phenotype is characterized by high IL-12 and IL-23 and low IL-10. (II) Tumor-promoting mutant M2 macrophages participate in the formation of tumor stroma; promote tumor growth, metastasis, and tumor angiogenesis; and lead to tumor immunosuppression. The phenotype is characterized by low IL-12 and IL-23 and high IL-10. Macrophages constitute the main cells of tumor inflammatory infiltration. There is a lineage of activated macrophages, and the difference between M1 and M2 phenotypes represents the extreme of this lineage (Mantovani et al., 2002). The polarization of M1 or M2 phenotype depends on the precise combination of signals in the local microenvironment. Macrophages change their functions and the expression of surface markers according to these changing signals (Figure 3). During persistent inflammation or in the TME, TAMs in established tumors are usually biased toward the M2 phenotype, which promotes the survival, development and spread of tumors by promoting angiogenesis, EMT and immunosuppression (Ye et al., 2015). The various tumor-prone functions of M2 macrophages create a microenvironment to support cell growth and immune evasion/inhibition, and promote the growth and development of tumors. Macrophages are closely linked and aggregated by activating key transcription factors in tumor cells, such as NF-κB, STAT3, and HIF-1α. Together, they lead to increased tumor cell proliferation, inhibition of apoptosis, neovascularization, ECM remodeling, migration, and invasion. Because macrophages are involved in multiple pathways of tumorigenesis, treatments for different stages of macrophages can be designed by inhibiting the recruitment, differentiation, and activation of macrophages, such as CCL2, COX-2, M-CSF, and GM-CSF, downregulating the PGE2/IL-6/STAT3 activation loop, and blocking the NF-κB signaling pathway (Hagemann et al., 2008; Coussens et al., 2013; Gutschalk et al., 2013; Mizuno et al., 2019; Piotrowski et al., 2020).

### Inflammatory Cytokines Associated With Lung Cancer

Cytokines link inflammation to tumor, and they are activated in both inflammatory cells and tumor cells. Cytokines play an important role in maintaining chronic inflammation, promoting the transformation of malignant epithelial cells, inhibiting tumor immune surveillance, and promoting tumor metastasis. The inflammatory factors closely related to lung cancer are IL-1β, IL-4, IL-6, IL-11, IL-12, TNF-α, MCP-1, and transforming growth factor (TGF)-β.

IL-1β: IL-1β is a member of the IL-1 cytokine family and is an important mediator of inflammatory response. It participates in a variety of cellular activities, including cell proliferation, differentiation, and apoptosis; the expression of IL-1 targets that promote tumor angiogenesis in chronic inflammation, and the expression of soluble mediators in cancer-related fibroblasts (CAFs) that cause antiapoptotic signals in tumor cells, thus promoting tumor progression (Bent et al., 2018). *In vitro* experiments showed that IL-1β induces angiogenesis and lymphatic angiogenesis *in vitro* (Nakao et al., 2011). IL-1β can increase tumor invasion and metastasis mainly by promoting angiogenic factors produced by stromal cells in the TME to induce tumor angiogenesis, endothelial cell activation, and immunosuppressive cells (Apte et al., 2006; Mantovani et al., 2018; Steel et al., 2018). Lee et al. found that IL-1β induces the expression of actin-binding protein fascin to promote tumor metastasis through the ERK1/2, JNK, NF-κB and CREB signal pathways (Lee et al., 2018). Weichand et al. found S1PR1 inflammatory bodies on TAMs promote lymph angiogenesis and metastasis through NLRP3/IL-1β (Weichand et al., 2017).

TNF-α: TNF-α is one of the most important cytokines produced by macrophages, the main mediator of cancer-related inflammation, and the key factor of inflammatory response, which was first proposed by Carswell et al. (Helson et al., 1975). Many pathogenic factors can induce TNF-α, which further induces other inflammatory mediators and proteases. During chronic inflammation, the imbalance and persistent production of TNF may promote carcinogenesis, and, in some cases, TNF may even be a carcinogen. An increasing number of evidences shows that a small amount of TNF-α produced by tumor cells and stromal cells is an endogenous tumor promoter (Balkwill, 2002). Tumor production of TNF-α is associated with poor prognosis, loss of hormone response, and cachexia (Kim et al., 2013). The NF-κB signal pathway is the bridge between TNF and tumor promotion (Greten et al., 2004). TNF produced by malignant cells can also cause excessive permeability of existing blood vessels, thus stimulating pleural effusion in lung cancer models (Stathopoulos et al., 2007). Preclinical studies have shown that the anti-tumor effect of TNF is due to the destruction of tumor vascular system (Daniel and Wilson, 2008). Outside the field of cancer, especially in rheumatism, TNF has been identified as a major regulator of inflammation and a key participant in the cytokine network, which has led to the development of antagonists of its role and revolutionized the treatment of rheumatoid arthritis and other inflammatory diseases (Tracey et al., 2008; Billmeier et al., 2016; Green et al., 2019). With the progress of research in patients with chronic inflammatory diseases, many mechanisms of TNF antagonist therapy in inhibiting TNF-enhanced cancer development have been found: angiogenesis, leukocyte infiltration, and stimulation of other cytokines and chemokines (Charles et al., 1999). TNF antagonists are safe for cancer patients (Madhusudan et al., 2004; Brown et al., 2008).

IL-6: IL-6, a powerful multipotent proinflammatory cytokine, plays a key role in host defense against pathogens and acute stress (Yao et al., 2014). IL-6–Janus kinase 2 (JAK)–signal transduction and transcriptional activator (STAT) signaling pathway plays an important role in various tumorigenesis models, including lung, breast, colon, ovarian, prostate, and multiple myeloma (Jin et al., 2013; Eskiler et al., 2019). CAFs isolated from human lung cancer tissue secrete IL-6, which stimulates JAK2 and STAT3 signal transduction in human lung cancer cells, thereby increasing metastasis (Wang L. et al., 2017). The combination of IL-6 blocking and other signal pathway inhibition has been widely studied in lung cancer. Caetano et al. found that IL-6 is overexpressed in a mouse model of K-ras mutant lung cancer and human lung cancer. The use of monoclonal anti-IL-6 antibody to block IL-6 can significantly reduce the promoting effect of COPD-like airway inflammation on lung tumor cell proliferation and tumor angiogenesis and bias the precursor immunosuppressive environment to anti-tumor phenotype, indicating that IL-6 is a potential drug target for the prevention and treatment of K-ras-mutant lung tumors. The study found that 8 (53%) of the 15 lung cancer cell lines expressed IL-6 mRNA and protein, suggesting that the overexpression of IL-6 may disrupt the cytokine balance and weaken the anti-tumor immunity of patients with lung cancer (Yamaji et al., 2004).

TGF-β: TGF-β is an evolutionarily conserved multipotent factor, which regulates many biological processes such as development, tissue regeneration, immune response, and tumorigenesis (Saito et al., 2018). It can promote tumor development by regulating certain microenvironmental pathways in a certain environment. It plays multiple roles in tumor biology and is often overexpressed in many tumors, including NSCLC (Bruno et al., 2013). High expression of TGF-β is a characteristic of NSCLC and a poor prognostic factor (Teixeira et al., 2011). High expression levels of TGF-β are related to lymph node metastasis and tumor angiogenesis in NSCLC (Hasegawa et al., 2001). William et al. found that the expression of TGF-β in lung adenocarcinoma is higher than that of other histological subtypes, which is the only independent immunological parameter with prognostic significance (Sterlacci et al., 2012). TGF-β also plays a role in the polarization of immune cells in the TME, including macrophages, neutrophils, and NK cells associated with tumor immune escape (Flavell et al., 2010). Some studies have shown that TGF-β-mediated EMT in cancer cells is related to antiapoptosis, acquisition of stem cell characteristics, chemotherapy resistance, and other invasive characteristics (Hao et al., 2019). TGF-β participates in the maintenance of T cell homeostasis and induces Treg cells that limit tumor immune response (Chen et al., 2003). Blocking TGF-β signal transduction with TGF-β-blocking antibodies or TGF-β receptor I kinase inhibitors can enhance anti-tumor immunity and show therapeutic benefits (Tauriello et al., 2018).

IL-10: IL-10 is a multifunctional cytokine with immunosuppressive and anti-angiogenesis functions that is mainly secreted by M2 macrophages, Treg cells, and Th2 cells. Polymorphisms in the IL-10 gene promoter region are associated with susceptibility to a variety of cancers (including lung cancer) (Namazi et al., 2018). Many patients with malignant tumors, including lung cancer, have elevated serum and peritumoral IL-10 levels (Vahl et al., 2017). Increased IL-10 mRNA expression is associated with the advanced lung cancer (Wang et al., 2011). The expression of IL-10 is a prognostic factor of NSCLC, and high IL-10 expression of TAMs is an important independent predictor of advanced tumor stage. Thus, it is related to poor overall survival rate. The local expression of IL-10 may promote tumor progression through the immunosuppression (Zeni et al., 2007).

MCP-1: MCP-1 is a chemokine that regulates monocyte chemotaxis and lymphocyte differentiation by binding to CC chemokine receptor 2 and plays a vital role in the pathogenesis of inflammatory diseases, atherosclerosis, and cancer (Bianconi et al., 2018). Many tumor lines and different non-tumor stromal cells in the TME, including fibroblasts, endothelial cells, and inflammatory cells, produce MCP-1, which can promote the proliferation, migration, and survival of tumor cells (Yoshimura, 2017). A meta-analysis evaluated the prognostic value of MCP-1 expression in patients with solid tumors, and the results showed that increased MCP-1 levels were associated with decreased overall survival (hazard ratio 1.95, 95% CI 1.32–2.88) (Wang et al., 2014). Studies have found that MCP-1 induces the interaction between tumor-derived factors and host-derived chemokines, thereby promoting bone metastasis (Mulholland et al., 2019).

### Inflammation-Related Signal Pathways of Associated With Lung Cancer

The signal pathways related to the inflammatory microenvironment mainly include NF-κB signal pathway and STAT3 signal pathway. Both pathways are involved in the process of inflammation and tumor production and are extremely active in various tumor cells. Thus, they are considered to be the most important participants in the tumor signal pathway.

**FIGURE 4:**
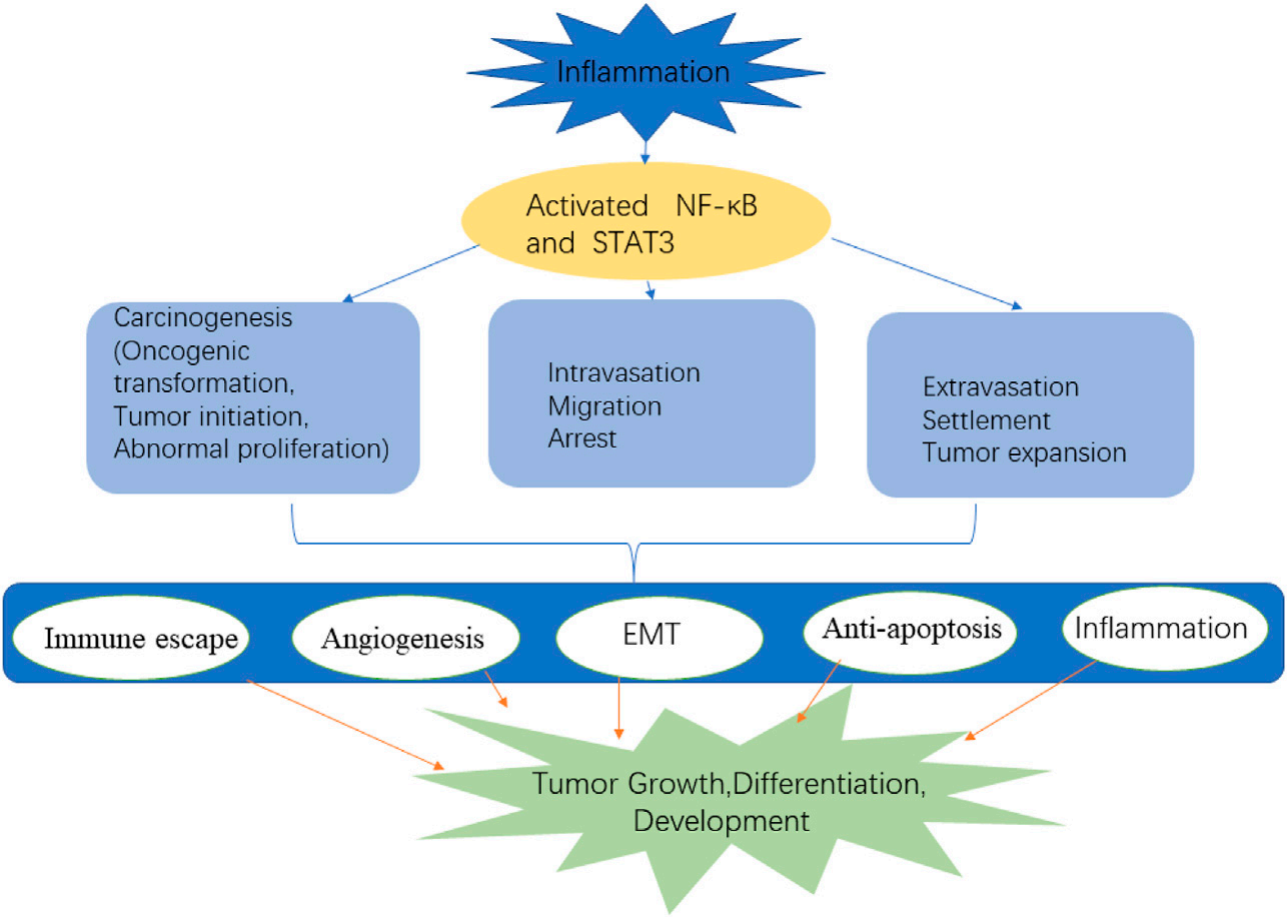
The multifaceted role of NF-κB and STAT3 in tumor.

NF-κB: NF-κB is a positive regulator of cell growth and proliferation, which is often activated by proinflammatory cytokines such as IL-1β and TNF-α. In most cases, NF-κB remains active in cancer cells through mutations in upstream signal molecules or in response to extracellular stimuli in the TME (Karin et al., 2002). NF-κB is structurally activated in a variety of solid tumors, including prostate cancer, breast cancer, cervical cancer, pancreatic cancer, and lung cancer. The NF-κB pathway is an important tumor signal pathway that plays an important role in the inflammatory response induced by lung cancer gene mutation (Lin et al., 2010). NF-κB promotes the key steps of tumor cell invasion and EMT, which is closely related to tumor apoptosis and angiogenesis (Figure 4). NF-κB increases the expression of several factors related to cell cycle progression, such as cyclin D and E (Chen et al., 2011). The upregulation of cyclin D1 expression by NF-κB is related to the increased transition from G1 phase to S phase. In addition, NF-κB negatively regulates the expression of growth arrest and DNA damage-inducible protein 45 (GADD45). GADD45 is a checkpoint protein of the cell cycle, which causes cells to transition in the G2/M phase. In addition, the interaction between NF-κB and proinflammatory cytokines such as TNF-α and IL-1β is also involved in stimulating cancer cell proliferation, especially during chronic inflammation (Rius et al., 2008). Although lung cancer is histologically heterogeneous, tumor samples obtained from lung cancer patients show a high level of NF-κB activation in both small cell lung cancer and NSCLC, which is significantly correlated with TNM stage and poor prognosis in these patients. The inhibition of NF-κB by siRNA, IKK inhibitors, and IκB super inhibitors can inhibit the survival and proliferation of lung cancer cells (Chen et al., 2011).

STAT3: Among the seven members of the STAT protein family (STAT1, STAT2, STAT3, STAT4, STAT5a, STAT5b, and STAT6), STAT3 and STAT5 are the most important factors for tumor progression (Yu and Jove, 2004). STAT3 plays a key role in the process of cell surface receptors transmitting extracellular signals to the nucleus. It is an important regulator of immunity, inflammation, and tumorigenesis and is the convergence point of a variety of signal transduction pathways (Dutta et al., 2014). The structural activation of STAT3 is involved in many cellular processes, including proliferation, survival, inflammation, invasion, metastasis, and angiogenesis, all of which are conducive to tumor initiation and progression (Siveen et al., 2014). In addition to its established role as a transcription factor in cancer, STAT3 regulates mitochondrial function and gene expression through epigenetic mechanisms (Yu et al., 2014). The activation of STAT3 is also an effective immune checkpoint for a variety of anti-tumor immune responses (Yu et al., 2009). Abnormal STAT3 signals promote the occurrence and development of a variety of human cancers by inhibiting apoptosis or inducing cell proliferation, angiogenesis, invasion, and metastasis (Grivennikov and Karin, 2010). STAT3 has been shown to prolong the survival of human PC-13 large cell lung cancer cells after serum deprivation (Akca et al., 2006). The structural activation of STAT3 is a common feature of NSCLC, and it is also considered to play an important role in tumor resistance to conventional and targeted small molecule therapy, especially the rich mutations in the JAK/STAT pathway (Govindan et al., 2012; Looyenga et al., 2012; You et al., 2014). In NSCLC cells, IL-6 neutralizing antibodies have been shown to inhibit tumor growth in mouse transplanted tumor models by inhibiting JAK1/STAT3 signaling (Song et al., 2011).

The NF-κB family and STAT3 are widely expressed, which can be activated rapidly under the stimulation of stress and cytokines. Once activated, NF-κB and STAT3 control the expression of antiapoptosis, proliferation, and immune response genes (Grivennikov and Karin, 2010). The activation and interaction of STAT3 and NF-κB play a key role in controlling the dialogue between tumor cells and their microenvironment, especially in inflammatory/tumor-infiltrating immune cells. They are powerful activators of malignant tumor state, affect the persistence of inflammation, and promote the production of tumor cytokines. They play an important role as a bridge between tumor cells and peripheral inflammatory cells. In the tumor inflammatory microenvironment, the activation of these two signal pathways can promote the release of corresponding cytokines, chemokines, and active substances, as well as the malignant proliferation and adhesion of tumor cells (Figure 4). Antiapoptotic genes are the main targets of NF-κB and STAT3, and Bcl-xL, B-cell lymphoma 2 (Bcl-2), Mcl-1, and other genes are activated by these two factors, especially can promote the expression of anti-apoptotic genes, such as Bc1-2 and so on (Rebouissou et al., 2009; Siveen et al., 2014). In addition, they can further chemotactic inflammatory cells by promoting the release of enzymes related to cytokines, chemokines, and prostaglandin synthesis and inducible nitric oxide synthase, thus forming an inflammatory microenvironment conducive to tumor production. In short, these two pathways play a key role in tumor cell production, survival, EMT, invasion, metastasis, inhibition of adaptive immunity and drug resistance.

## Mechanisms of Tumor Inflammatory Microenvironment in Promoting the Development and Metastasis of Lung Cancer

The TME has been considered as a major factor in tumor progression and metastasis (Hanahan and Coussens, 2012). Its composition is heterogeneous. The coordinated heterogeneous interaction between genetically altered tumor epithelial cells and tumor stromal cells regulates the main characteristics of the tumor, including immune escape, angiogenesis, EMT, and metastasis. Myeloid cells (monocytes, macrophages, and neutrophils) also secrete VEGF, basic fibroblast growth factor, platelet-derived growth factor, placental growth factor, and Bv8, all of which contribute to vascular remodeling during tumor progression (Shojaei et al., 2007). The major mechanisms of the inflammatory microenvironment in promoting lung cancer metastasis are as follows.

### Immune Escape

More and more attention has been paid to the role of the immune system in tumorigenesis, and immune escape is now regarded as one of the markers of cancer. Abnormalities in the structure and function of the immune system are closely related to the occurrence and development of tumors. There are complex interactions between immune cells and malignant cells in the tumor stroma. The immune system can not only promote the tumor, but also inhibit it. On the one hand, the body’s immune system can sense the existence of tumors and kill or eliminate tumor cells through a variety of immune mechanisms. On the other hand, tumor cells can also escape or resist the killing and clearance of tumor cells by the immune system through a variety of mechanisms. Burnet put forward the concept of immune surveillance in 1970: the immune system spontaneously recognizes and eliminates cancer cells, thus preventing tumor development (Burnet, 1970). Inflammatory cells and inflammatory factors can help tumor cells escape immune surveillance. Many factors released by inflammatory cells may directly or indirectly lead to significant inhibition of immune response (Ben-Baruch, 2006). For example, chronic tumor cells secrete cytokines and other soluble factors and subsequently induce, expand, and recruit Treg cells to tumor sites. A high proportion of Treg cells produce an immunosuppressive microenvironment, which inhibits anti-tumor immunity and promotes tumor growth. TAMs further induce immunosuppression by activating immune checkpoint PD-L1 and increasing the expression of specific metabolic pathways arginase-1 and indoleamine 2,3-dioxygenase (Johnson and Munn, 2012).

### Tumor Angiogenesis

Angiogenesis plays a key role in the growth, proliferation, and metastasis of various solid tumors (Rivas-Fuentes et al., 2015). Pathological angiogenesis is a hallmark of cancer and various ischemic and inflammatory diseases, and tumor tissues of lung cancer show active angiogenesis. Chronic inflammation is related to angiogenesis, which is a process that helps cancer cells grow. Studies have shown that different types of cells in the TME can affect angiogenesis, participate in the regulation of secreted local concentrations of pro-angiogenic factors and anti-angiogenic factors, and change the local insoluble matrix surrounding blood vessels. Among them, inflammatory infiltrating cells (including monocytes, macrophages, mast cells) and inflammatory factors are involved in the regulation of angiogenesis. Inflammation-related chemokines (including CC, CXC, CX3C, and XC family) play a central role as tumor angiogenesis regulators. In turn, the endothelial cells that form the vascular system actively participate in and regulate the inflammatory response of normal and diseased tissues (Pober and Sessa, 2007).

### Epithelial-To-Mesenchymal Transition

The concept of EMT comes from the study of events related to development (Shook and Keller, 2003). Tumor metastasis begins when cancer cells infiltrate from the epithelial layer to adjacent tissues and at least temporarily acquire the EMT phenotype, which enables cancer cells to move and penetrate the basement membrane, invade the tissues, and reach the lymphatics or blood vessels for further spread (Varga and Greten, 2017). Inflammation is an effective cause of EMT in tumors, and the EMT program can also stimulate cancer cells to produce proinflammatory factors. Therefore, inflammation and EMT are inseparable factors in cancer progression (Suarez-Carmona et al., 2017). In the TME, TGF-β, CXCL4/12, IL-6, and TNF-α can enhance EMT. At the same time, tumor cells secrete more epithelial growth factors, fibroblast growth factors, and insulin-like growth factor, which lead to low oxygen, acidity, and high interstitial fluid pressure state in the microenvironment and activates CAFs to produce more matrix metalloproteinases and reshape the tumor ECM (Jung et al., 2015). A previous study found the weighted score of tumor inflammatory signals and the genetic characteristics of EMT can accurately predict the response of lung cancer patients to immune checkpoint blockade (Thompson et al., 2019). In lung cancer cells, erlotinib-induced autocrine IL-8 production can induce EMT and trigger the drug resistance of erlotinib through the p38MAP kinase pathway (Fernando et al., 2016).

### Anti-apoptosis

Apoptosis is the most common form of programmed cell death in vertebrates and one of the key determinants in regulating the efficiency of tumor metastasis. It is generally considered to be an important mechanism for negative regulation of cancer development. Apoptotic pathways include external apoptotic pathways (dependent on death receptors) and internal apoptotic pathways (dependent on mitochondria). The intrinsic pathway is closely regulated by the intracellular protein Bcl-2 family. The exogenous apoptosis pathway is initiated by the binding of ligands (Fas-related death domains) to death receptors (death-inducing signal complexes) that contain intracellular death domains. Intrinsic pathways are activated by physical or chemical stimuli, such as hypoxia, growth factor deprivation, cell detachment, or stress signals (Millimouno et al., 2014).

**FIGURE 5:**
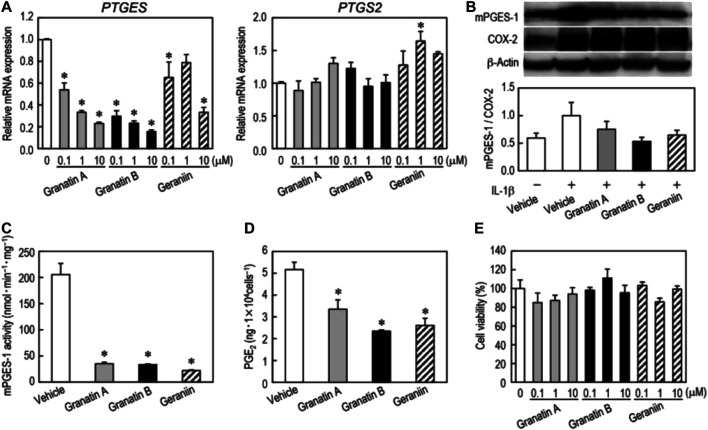
Effects of granatin A, granatin B, and geraniin on mPGES-1 and COX-2 expression. Changes in mRNA and protein levels of mPGES-1 and COX-2 by treatment with granatin A, granatin B, and geraniin were analyzed by real-time PCR and western blotting. Reproduced from Toda et al., 2020 with permission from Taylor and Francis Ltd. Copyright 2019.

Toshiko et al. found that granatin A and granatin B, ellagitannins isolated from pomegranate leaves, and geraniin, their structural analog, could selectively suppress mPGES-1 expression without affecting COX-2 in non-small cell lung carcinoma A549 cells (Figure 5). The ellagitannins also downregulated TNF-α, inducible nitric oxide synthase, and antiapoptotic factor Bcl-2, and induced A549 cells to undergo apoptosis (Toda et al., 2020). Padwad et al. found that berberine can induce dose-dependent resting and apoptosis of A549 cancer cells by regulating cyclin and inflammation independent of the mTOR pathway (Kumar et al., 2020). The IL-33/ST2 axis can modify the TME to support malignant proliferation or enhance anti-tumor immunity by recruiting immune cells. It is also responsible for the release of NF-κB, thus increasing the expression of GLUT1 in NSCLC. The administration of anti-IL-33 and anti-ST2 drugs effectively limit this process and reduce the presence of Treg cells in cancer sites (Wang K. et al., 2017).

## Progress in the Treatment of Lung Cancer Based on Inflammation and Inflammatory Microenvironment

**FIGURE 6:**
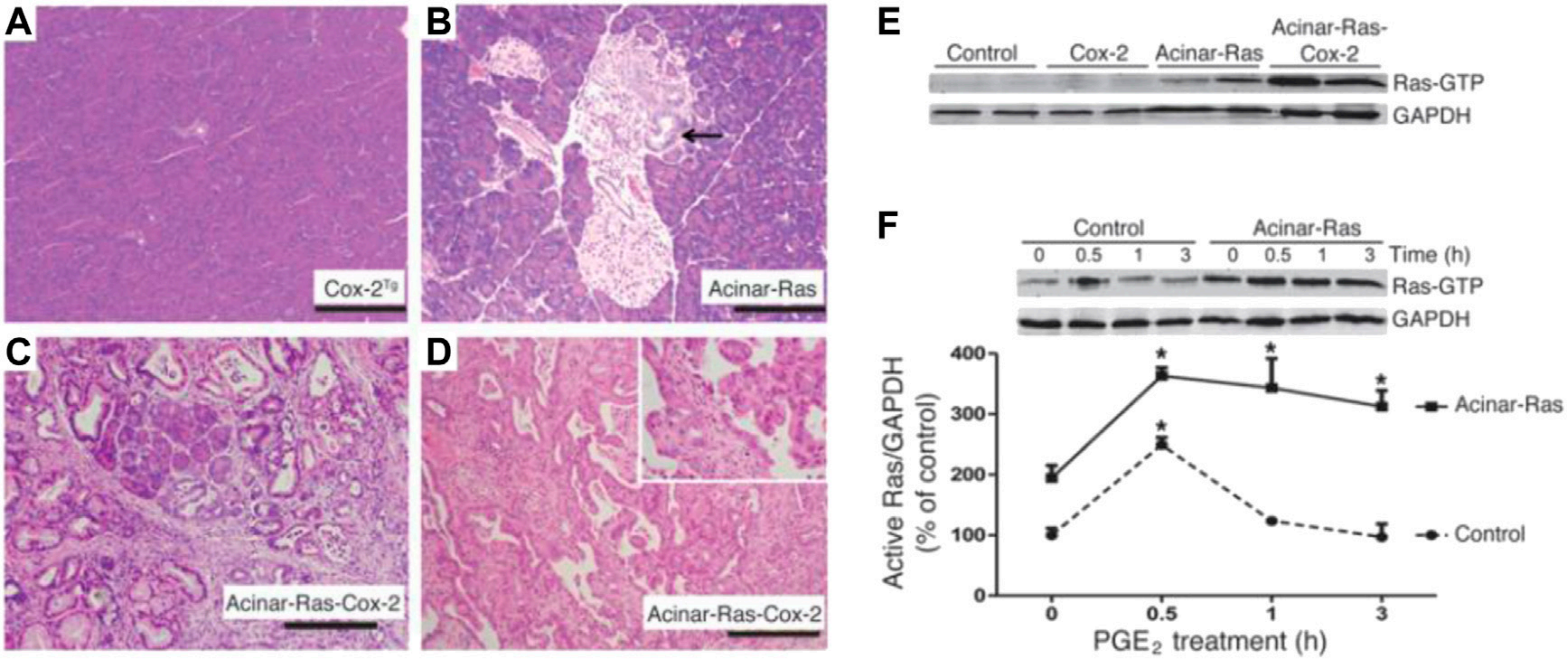
COX-2 and oncogenic K-Ras synergized to promote the development of chronic inflammation and cancer. Reproduced from Daniluk et al., 2012 with permission from JCI. Copyright 2012.

The treatment of lung cancer has developed from specific cytotoxic drugs to more specific molecular targeted drugs. Although great progress has been made in the treatment of lung cancer with the development of targeted therapy and immune therapy, the 5-years survival rate of NSCLC is still about 15%, and most lung cancer patients do not show targeted gene mutations (Gettinger et al., 2018). Traditional surgery, radiotherapy, and chemotherapy are still the only treatment options for most advanced NSCLC patients, but these treatments are unsatisfactory (Choi et al., 2010). Consequently, more and more studies have begun to investigate lung cancer from the perspective of the TME, especially inflammation; actively explore targets related to anti-angiogenesis, immune regulation, and EMT in the microenvironment; and develop more therapeutic targets (Blumenschein, 2012). For example, Logsdon et al. found that in the presence of carcinogenic Ras, inflammatory stimulation initiates a positive feedback loop involving NF-κB, which further amplifies the activity of Ras to the pathological level (Figure 6). The effect of these inflammatory stimuli can be blocked through the deletion of NF-κB kinase 2 inhibitor or the inhibition of COX-2. Because a large number of lung cancer patients have Ras gene mutations, blocking this positive feedback loop may be an important strategy for cancer prevention (Daniluk et al., 2012).

Epidemiological research and meta-analysis showed that long-term use of aspirin, a non-steroidal anti-inflammatory drug (NSAID), can reduce the risk of various solid tumors including lung cancer, and taking NSAIDs every day for 1 or 2 years can reduce the relative risk of lung cancer by 60–68% (Smith et al., 2006). The chemoprevention characteristics of the long-term use of NSAIDs are based on their cyclooxygenase inhibitory activity, and the ability to inhibit COX-1 and COX-2 is the basis of NSAIDs’ chemoprevention mechanism. In some cancers (e.g., NSCLC), the overexpression of COX-2 is associated with poor prognosis (Lee et al., 2008). COX-2 is closely related to apoptosis resistance, angiogenesis, decreased host immune function, invasion, and metastasis and plays a key role in the occurrence and development of tumors. However, many NSAIDs, especially those that selectively inhibit COX-2, can cause life-threatening adverse effects such as gastric ulcers, heart attacks, and stroke in a number of patients (Singh et al., 2019).

## Future Outlook and Conclusion

Signal molecules in the inflammatory microenvironment have extensive effects on the maintenance and development of lung cancer. More and more animal and human experiments have revealed that lung cancer can be regulated by interfering with inflammation and inflammatory signal pathway. The in-depth understanding of the molecular mechanisms of inflammatory cells and tumor growth, angiogenesis, and progression in the TME will help to identify new therapeutic targets and design new drugs without serious side effects. It will also help promote the development of anti-tumor vaccines and other therapeutic strategies.
